# Steroidal Saponins from the Genus *Smilax* and Their Biological Activities

**DOI:** 10.1007/s13659-017-0139-5

**Published:** 2017-06-23

**Authors:** Li-Wen Tian, Zhen Zhang, Hai-Lan Long, Ying-Jun Zhang

**Affiliations:** 1 School of Pharmaceutical Sciences, Southern Medical University, Guangzhou, 510515 China; 2 State Key Laboratory of Phytochemistry and Plant Resources in West China, Kunming Institute of Botany, Chinese Academy of Sciences, Kunming, 650201 China

**Keywords:** *Smilax*, Steroidal saponins, Biological activities

## Abstract

**Abstract:**

The *Smilax* species, widely distributed in tropical region of the world and the warm areas of East Asia and North America, are extensively used as folk medicine to treat inflammatory disorders. Chemical investigation on *Smilax* species showed they are rich sources of steroidal saponins with diversified structure types, including spirostane, isospirostane, furostane, pregnane, and cholestane. This review mainly summarizes the steroidal saponins (**1**–**104**) reported from the genus *Smilax* between 1967 and 2016, and their biological activities. The relationship between structures of steroidal saponins and related biological activities were briefly discussed.

**Graphical Abstract:**

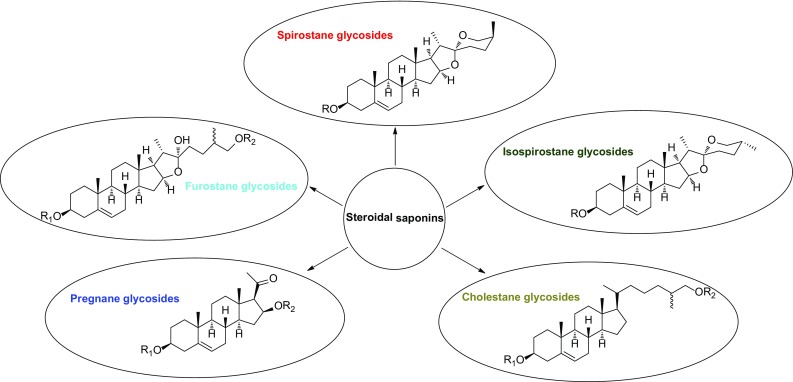

## Introduction

The genus *Smilax* (Liliaceae family) comprises about 300 species of climbing shrubs. Plants of the genus are widely distributed in tropical region of the world, and also found in warm areas of East Asia and North America [1]. The juvenile leaves of *S. riparia* are used as vegetable product. The rhizomes of *S. glabra* are used in Southeast of China as food supplementary for health. Noteworthily, the rhizomes of *Smilax* species are most famous for their medical use. The rhizomes of *S. china* and *S. glabra*, called “Jin Gang Teng” and “Tu Fu Lin” in Pharmacopoeia of People’s Republic of China respectively, are clinically used to treat chronic pelvic inflammatory disease, rheumatic arthritis and so on [2]. The rhizomes of *S. riparia*, *S. nipponica*, *S. bockii*, *S. microphylla*, and *S. discotis* were recorded in the Chinese Herbal Medicines to treat joint pain, edema, and rheumatoid arthritis [3].

Previous studies on chemical constituents of *Smilax* species have disclosed the presence of steroidal saponins, flavonoids, phenylpropanoids, and stilbenoids [4]. Astilbin, a main flavonoid among *Smilax* species [5], showed unique immunosuppressive activity, and proved to be the active material basis of *Smilax* species for the treatment of human immune diseases [6]. Steroidal saponins are characteristic bioactive components of the genus *Smilax* in terms of chemotaxonomic value and biological activities [7]. So far, 104 steroidal saponins have been reported from 20 different *Smilax* species. These steroidal saponins showed significant antifungal, cytotoxic, anti-inflammatory, as well as cAMP phosphodiesterase inhibitory activities.

In this review, steroidal saponins reported from the genus *Smilax* between 1967 and 2016 were listed, and the biological activities of steroidal saponins were also included.

## Chemistry of Steroidal Saponins

**Fig. 1:**
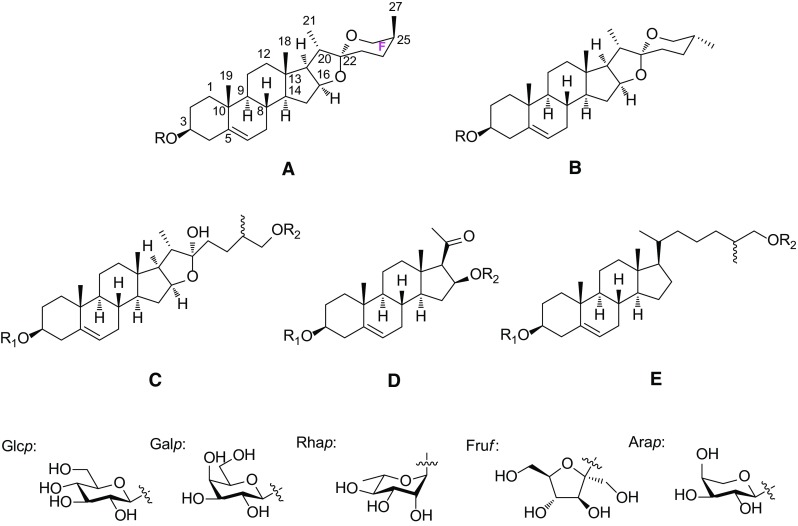
Structures of **a** a spirostane backbone, **b** an isospirostane backbone, **c** a furostane backbone, **d** a pregnane backbone, **e** a cholestane backone, a glucopyranosyl moiety (Glc*p*), a galactopyranosyl moiety (Gal*p*), a rhamnopyranosyl moiety (Rha*p*), a frutofuranosyl moiety (Fur*f*) and an arabinopyranosyl moiety

Steroidal saponins from the genus *Smilax* could be divided into five groups on the basis of the sapogenin structures: spirostane (A), isospirostane (B), furostane (C), pregnane (D), and cholestane (E) (Fig. 1). They are mostly mono- or bisdesmosides. A carbohydrate chain is always attached to the C-3 position of sapogenin by an ether linkage. Additionally, C-26 position of furostane-type saponin is always etherified with a glucopyranosyl moiety. So far only one steroidal saponin from the genus *Smilax*, (25*S*)-26-*O*-*β*-d-glucopyranosyl-5*β*-furostan-1*β*,3*β*,22*α*,26-tetraol-1-*O*-*β*-d-glucopyranoside (**92**), has a glucopyranosyl moiety linked to the C-1 position. The sugar residues consist of linear or branched saccharidic chains, made up most often of glucopyranosyl (Glc*p*), rhamnopyranosyl (Rha*p*), galactopyranosyl (Gal*p*), fructofuronosyl (Fru*f*), and arabinopyranosyl (Ara*p*) moieties (Fig. 1).

### Spirostane-Type Saponins **1**–**11**

**Fig. 2:**
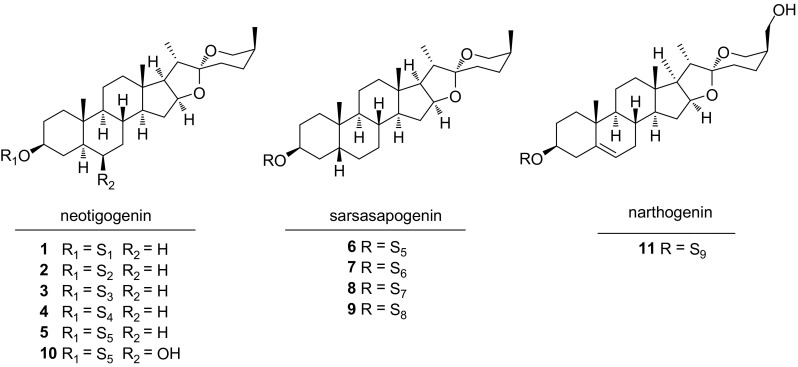
Structures of compounds **1**–**11**

Spirostane-type saponins are monodesmosidic glycosides with six rings A–F in sapogenin. They are characterized by an axial oriented methyl or hydroxymethyl (C-27) on F ring. The sapogenin of spirostane glycosides **1**–**11** possess either a *cis* or a *trans* fusion between rings A and B, or a double bond between C-5 and C-6, leading to 5*α* (neotigogenin), 5*β* (sarsasapogenin), and Δ^5^ (narthogenin) subtypes (Fig. 2). Neotigogenin glycosides **1**–**5**, and **10** have been isolated from *S. riparia* [8], *S. nipponica* [9], and *S. officinalis* [7]. Both neotigogenin glycosides **5**, **10** and sarsasapogenin glycoside **6** were identified from the rhizomes of *S. officinalis* [7]. Sarsasapogenin glycosides **7**–**9** were isolated from the root of *S. aspera* subsp. *mauritanica* [10], and *S. ornata* Lem. [11]. Compound **11**, with a hydroxyl substitution on C-27, was the only narthogenin glycoside reported from *Smilax* species so far.

### Isospirostane-Type Saponins **12**–**47**

**Fig. 3:**
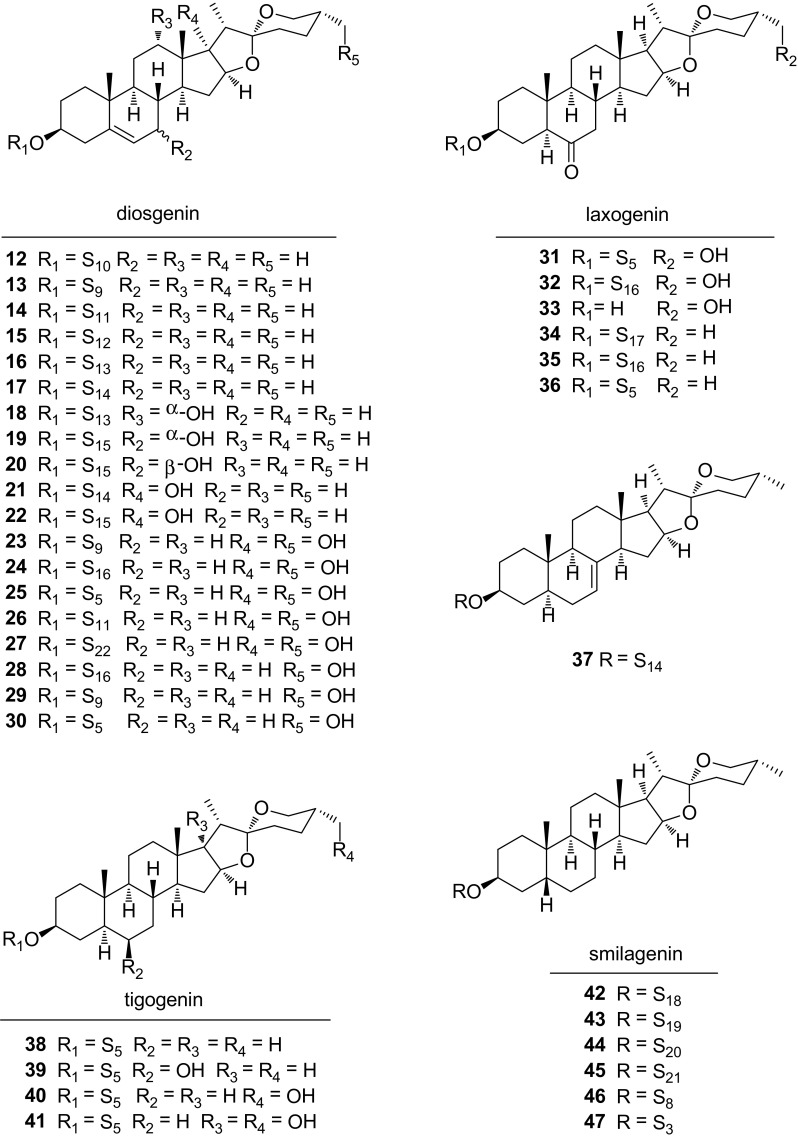
Structures of compounds **12**–**47**

Isospirostane-type saponins are also monodesmosidic glycosides characterized by an equatorial oriented methyl or hydroxymethyl (C-27) on F ring. The isospirostane-type saponins **12**–**47** could be classified into four subtypes on the basis of sapogenin structures, including diosgenin, laxogenin, tigogenin, and smilagenin (Fig. 3). The variations of these sapogenins mainly comprise dehydrogenation between C-5 and C-6, carbonylation at C-6, hydroxylation at C-17 or C-27, and *cis*/*trans* fusion between rings A and B. Diosgenin glycosides **12**–**30** were characterized by a double bond between C-5 and C-6. Diosgenin-3-*O*-*α*-l-rhamnopyranoside (**12**) was the first diosgenin glycoside reported from the epigeal part of *S. excelsa* in 1975 [12]. Dioscin (**13**) was widely distributed among the *Smilax* species, including *S. china* [8], *S. menispermoidea* [13], *S. lebrunii* [14], *S. nigrescens* [15], *S. stans* [16], *S. excels* [17], *S. microphylla* [18], and *S. bockii* [19]. Parisyunnanosides C–E (**18**–**20**), with hydroxyl substitutions at C-7 or C-12, were isolated from the stems of *S. riparia* [20]. The occurrences of parisyunnanoside in the genus *Smilax* indicated the close chemtaxonomic relationship between the genus *Smilax* and *Paris*. Three isonarthogenin glycosides **24**, **25**, and **28** were isolated from *S. scobinicaulis*, together with two tigogenin glycosides **38**–**39** [21]. Sieboldogenin (**33**), with an additional hydroxyl substitution on C-27 in comparison with laxogenin, was identified from the ethyl acetate fraction of *S. china* [22]. Laxogenin glycosides **34**–**36** were founded in *S. sieboldii* [23]. Parisvietnaside A (**37**), characterized by a double bond between C-7 and C-8, was obtained from the roots and rhizomes of *S. riparia* [24]. The smilagenin glycosides **42**–**47** with a *cis* fusion rings A and B were isolated from the roots of *S. medica* [25, 26]. Hydroxyl substitution on C-7 or C-12, and double bond between C-7 and C-8 are the rare cases within the steroidal saponins of the genus *Smilax*.

**Fig. 4:**
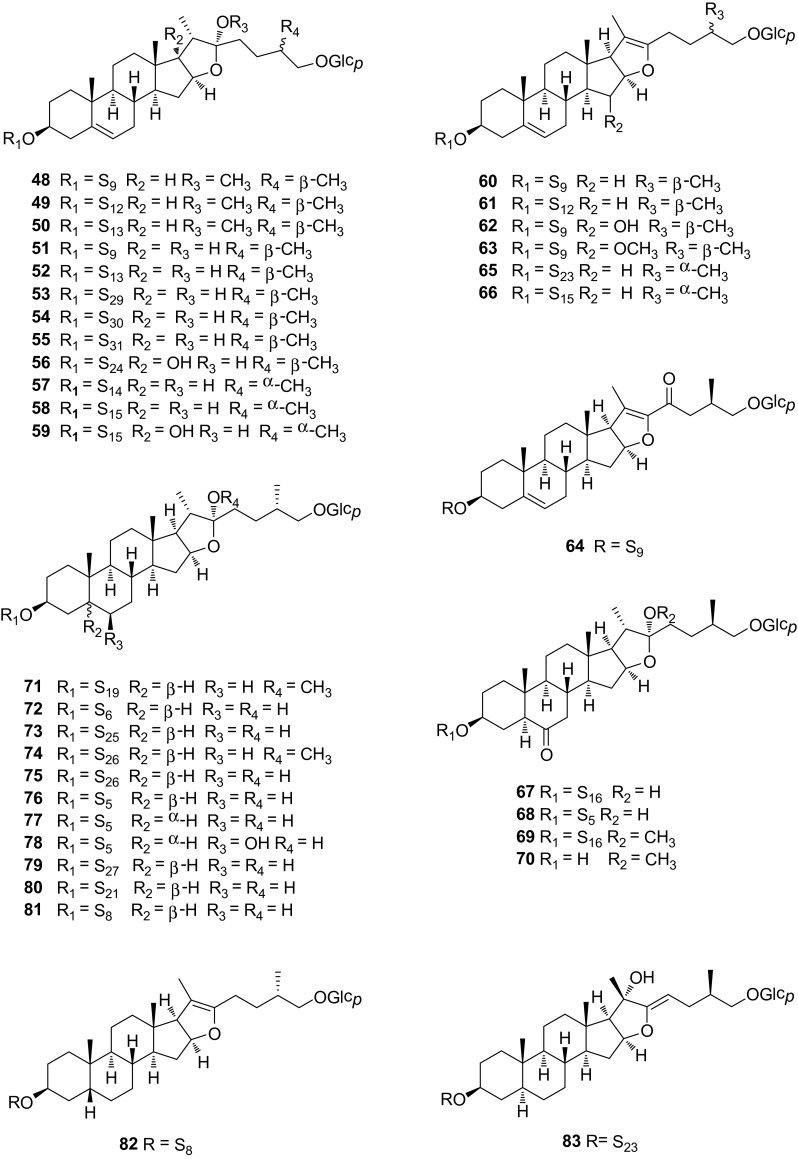
Structures of compounds **48**–**83**

### Furostane-Type Saponins **48**–**93**

Furostane-type saponins, F ring opened spirostanol glycosides, are another important group of steroidal saponins within *Smilax* species. The hemiketal hydroxy attached to the C-22 position of furostanol glycosides were sometimes methylated or dehydrated. The methylated derivatives were generally considered to be artifacts. Furostanol glycosides with both 25*R* and 25*S* configurations were reported from the genus *Smilax*. Additionally, furostanol glycosides always have two sugar chains attached to the C-3 and C-26 positions of the aglycone moiety (Fig. 4). Methylprotodioscin (**48**), protodioscin (**59**), and pseudoprotodioscin (**60**) were common constituents among the different *Smilax* species (Table 1). Compounds **50**, isolated from the roots of *S. bockii*, increased the nerve growth factor (NGF)-induced neurite outgrowth in PC 12D cells by 49% in comparison with the blank control at the concentration of 60 μg/mL [19]. Compounds **53**–**55**, identified from the rhizomes of *S. excelsa*, were the only three steroidal saponins with acylated sugars moieties within the genus *Smilax* [17]. Furostane glycosides **62** and **63**, with an oxygenated C-15, were isolated from the tubers of *S. china* [27]. Interestingly, the spirostane or isospirostane glycosides with an oxygenated C-15 have never been reported from *Smilax* so far. Compounds **67**–**70** with carbonylation on C-6 were isolated from the roots and rhizomes of *S. scobinicaulis*, together with a spirostane glycoside **35**, and three furostane glycosides **89**–**91** [28]. Compounds **76** and **77**, isolated from the root of *S. officinalis*, are the diastereoisomers with opposite configuration at C-5 [29]. Smilaxosides A–C (**84**, **86**, **87**), and (25*R*)-Smilaxchinoside A (**85**) were obtained tubers from *S. china* [30]. Of them, compounds **84** and **85** are diastereoisomers with opposite configuration at C-25. Compounds **92** and **93**, identified from *S. aspera* [31], were rare examples with hydroxyl substitution on C-1 within the genus *Smilax*.

**Table 1:** Steroidal saponins from the Genus *Smilax*

No.	Name	Plant	Parts	Ref.
*Spirostane-type saponin*				
1	Neotigogenin-3-*O*-*α*-l-rhamnopyranosyl-(1 → 6)-*β*-d-glucopyranoside	*S. riparia* *S. lanceaefolia*	Rhizomes and roots Roots	[8] [37]
2	Neotigogenin-3-*O*-*β*-d-glucopyranosyl-(l → 4)-*O*-[*α*-l-rhamnopyranosyl-(l → 6)]-*β*-d-glucopyranoside	*S. riparia*	Rhizomes and roots	[8]
3	Neotigogenin-3-*O*-*β*-d-glucopyranoside	*S. nipponica*	Subterranean	[9]
4	Smilanippin A	*S. nipponica*	Subterranean	[9]
5	Neotigogenin-3-*O*-*β*-d-glucopyranosyl-(1 → 4)-*O*-[*α*-l-arabinopyranosyl-(1 → 6)]-*β*-d-glucopyranoside	*S. officinalis*	Rhizomes	[7]
6	Sarsasapogenin-3-*O*-*β*-d-glucopyranosyl-(1 → 4)-[*α*-l-arabinopyranosyl- (1 → 6)]-*β*-d-glucopyranoside	*S. officinalis*	Rhizomes	[7]
7	(25S)-5*β*-Spirostane-3*β*-ol-3-*O*-*α*-l-rhamnopyranosyl-(1 → 2)-*β*-d-glucopyranosyl-(1 → 2)-*β*-d-glucopyranoside	*S. aspera* subsp. *mauritanica*	Roots	[10]
8	Curillin G	*S. aspera* subsp. *mauritanica*	Roots	[10]
9	Parillin	*S. aristolochiifolia* *S. ornate*	Rhizomes and roots	[38] [11]
10	(25*S*)-Spirostan-6*β*-ol-3-*O*-*β*-d-glucopyranosyl-(1 → 4)-*O*-[*α*-l-arabinopyranosyl-(1 → 6)]-*β*-d-glucopyranoside	*S. officinalis*	Rhizomes	[7]
11	Trinervuloside C	*S. trinervula*	Rhizomes and roots	[32]
*Isospirostane-type saponin*				
12	Diosgenin-3-*O*-*α*-l-rhamnopyranoside	*S. excels*	Epigeal part	[12]
13	Dioscin	*S. china* *S. menispermoides* *S. lebrunii* *S. nigrescens* *S. stans* *S. bockii* *S. excelsa* *S. microphylla* *S. china*	Roots Rhizomes Roots Roots Roots Roots Rhizomes Tubers Tubers	[8] [13] [14] [15] [16] [19] [17] [18] [30]
14	Diosgenin-3-*O*-[*α*-l-rhamnopyranosyl-(l → 4)]-*β*-d-glucopyranoside	*S. nigrescens* *S. menispermoides* *S. menispermoides* *S. china*	Roots Roots Rhizomes Tubers	[15] [39] [33] [30]
15	Diosgenin-3-*O*-[*α*-l-rhamnopyranosyl-(l → 2)]-*β*-d-glucopyranoside	*S. nigrescens* *S. menispermoides* *S. microphylla*	Roots Rhizomes Tubers	[15] [33] [18]
16	(25*R*)-Spirostan-5-en-3-*O*-*α*-l-rhamnopyranosyl-(l → 2)-[*α*-l-rhamnopyranosyl-(l → 4)-*α*-l-rhamnopyranosyl-(l → 4)]-*O*-*β*-d-glucopyranoside	*S. china*	Tubers	[30]
17	Gracillin	*S. microphylla*	Tubers	[18]
18	Parisyunnanoside C	*S. riparia*	Rhizomes and roots	[20]
19	Parisyunnanoside D	*S. riparia*	Rhizomes and roots	[20]
20	Parisyunnanoside E	*S. riparia*	Rhizomes and roots	[20]
21	Paris D	*S. riparia*	Rhizomes and roots	[20]
22	Paris H	*S. riparia*	Rhizomes and roots	[20]
23	(25*R*)-Spirost-5-en-3*β*,17*α*,27-triol-3-*O*-*α*-l-rhamnopyranosyl-(l → 2)-[*α*-l-rhamnopyranosyl-(l → 4)]-*β*-d-glucopyranoside	*S. menispermoides*	Rhizomes	[40]
24	(25*S*)-Spirostan-5-en-3*β*,17*α*,27-triol-3-*O*-*α*-l-arabinopyranosyl-(1 → 6)-*β*-d-glucopyranoside	*S. lebrunii* *S. lebrunii* *S. scobinicaulis*	Roots Roots Rhizomes and roots	[14] [41] [21]
25	(25*S*)-Spirostan-5-en-3*β*,17*α*,27-triol-3-*O*-*β*-d-glucopyranosyl-(1 → 4)-[*α*-l-arabinopyranosyl-(1 → 6)]-*β*-d-glucopyranoside	*S. lebrunii* *S. lebrunii* *S. scobinicaulis* *S. scobinicaulis*	Rhizomes Rhizomes Rhizomes and roots Rhizomes	[33] [42] [21] [43]
26	(25*S*)-spirost-5-ene-3*β*,17*α*,27-triol-3-*O*-*α*-l-rhamnopyranosyl-(l → 4)-*β*-d-glucopyranoside	*S. menispermoides* *S. menispermoides*	Roots Rhizomes	[39] [33]
27	(25*S*)-Spirost-5-en-3*β*,17*α*,27-triol-3-*O*-*β*-d-galactopyranoside	*S. menispermoides*	Rhizomes	[33]
28	(25*S*)-Spirostan-5-en-3*β,*27-diol-3-*O*-*α*-l-arabinopyranosyl-(1 → 6)-*β*-d- glucopyranoside	*S. scobinicaulis* *S. lebrunii*	Rhizomes and roots Roots	[21] [14]
29	Isonarthogenin3-*O*-*α*-l-rhamnopyranosyl-(l → 2)-*O*-[*α*-l-rhamnopyranosyl-(l → 4)]-*β*-d-glucopyranoside	*S. china* *S. china*	Roots Tubers	[8] [30]
30	Smilscobinoside A	*S. scobinicaulis*	Rhizomes and roots	[44]
31	Sieboldiin A	*S. sieboldii* *S. sieboldii* *S. scobinicaulis*	Subterranean Rhizomes Rhizomes	[45] [23] [46]
32	Sieboldiin B	*S. sieboldii* *S. sieboldii* *S. scobinicaulis* *S. scobinicaulis*	Subterranean Rhizomes Rhizomes Rhizomes and roots	[45] [23] [46] [28]
33	Sieboldogenin	*S. china*	Rhizomes	[22]
34	(25*R*)-5*α*-Spirostan-3*β*-ol-6-one-3-*O*-[*α*-l-arabinopyranosyl-(l → 4)]-*β*-d-glucopyranoside	*S. lebrunii* *S. lebrunii*	Roots Roots	[14] [41]
35	Smilaxin A	*S. sieboldii* *S. lebrunii* *S. scobincaulis*	Subterranean Rhizomes Rhizomes	[45] [47] [48]
36	Smilaxin B	*S. sieboldii* *S. lebrunii* *S. sieboldii* *S. scobincaulis*	Subterranean Rhizomes Rhizomes Rhizomes	[45] [47] [23] [48]
37	Parisvietnaside A	*S. riparia*	Rhizomes and roots	[20]
38	Smilaxin C	*S. sieboldii* *S. sieboldii* *S. scobinicaulis*	Subterranean Rhizomes Rhizomes and roots	[45] [23] [21]
39	(25*R*)-5*α*-Spirostan-3*β,*6*β*-diol-3-*O*-*β*-d-glucopyranosyl-(1 → 4)-[*α*-l-arabinopyranosyl-(1 → 6)]-*β*-d-glucopyranoside	*S. scobinicaulis*	Rhizomes and roots	[21]
40	Smilscobinoside B	*S. scobinicaulis*	Rhizomes and roots	[44]
41	(25*R*)-5α-Spirostan-3*β,*17*α,*27-triol-3-*O*-*β*-d-glucopyranosyl-(1 → 4)-[*α*-l-arabinopyranosyl-(1 → 6)]-*β*-d-glucopyranosie	*S. scobinicaulis* *S. scobinicaulis*	Rhizomes Rhizomes and roots	[43] [44]
42	(25*R*)-5*β*Spirostan-3*β*-ol-3-*O*-*β*-d-glucopyranosyl-(1 → 6)-[*β*-d-glucopyranosyl-(1 → 4)]-*β*-d-glucopyranoside	*S. medica*	Rhizomes	[25]
43	(25*R*)-5*β*-Spirostan-3*β*-ol-3-*O*-*β*-d-glucopyranosyl-(1 → 6)-[*β*-d-glucopyranosyl-(1 → 2)]-[*β*-d-glucopyranosyl-(1 → 4)]-*β*-d-glucopyranoside	*S. medica*	Rhizomes	[25]
44	Disporoside A	*S. medica*	Rhizomes	[25]
45	(25*R*)-5*β*-Spirostan-3*β*-ol-3-*O*-*β*-d-glucopyranosyl-(1 → 6)-*β*-d-glucopyranoside	*S. medica*	Rhizomes	[26]
46	(25*R*)-5*β*-Spirostan-3*β*-ol-3-*O*-*β*-d-glucopyranosyl-(1 → 6)-[*β*-d-glucopyranosyl-(1 → 2)]-[*α*-l-rhamnopyranosyl-(l → 4)]-*β*-d-glucopyranoside	*S. medica*	Rhizomes	[26]
47	Smilagenin 3-*O*-*β*-d-glucopyranoside	*S. medica*	Rhizomes	[26]
*Furostane-type saponin*				
48	Methylprotodioscin	*S. china* *S. menispermoides* *S. stans* *S. bockii* *S. microphylla* *S. china* *S. nigrescens*	Roots Rhizomes Roots Roots Tubers Tubers Roots	[8] [13] [16] [19] [18] [30] [49]
49	26-*O*-*β*-d-Glucopyranosyl-(25*R*)-furostan-5-en-3*β*,26-diol-22-methoxy-3-*O*-*α*-l-rhamnopyranosyl-(l → 2)-*β*-d-glucopyranoside	*S. nigrescens*	Roots	[49]
50	26-*O*-*β*-d-Glucopyranosyl-22*α*-*O*-methyl-(25*R*)-furost-5-en-3*β,*26-diol-3-*O*-*α*-l-rhamnopyranosyl-(l → 4)-*α*-l-rhamnopyranosyl-(l → 4)-[*α*-l-rhamnopyranosyl-(l → 2)]-*β*-d-glucopyranoside	*S. bockii*	Roots	[19]
51	Protodioscin	*S. excelsa* *S. microphylla* *S. china*	Rhizomes Tubers Tubers	[17] [18] [30]
52	Protodiosgenin-3-*O*-*α*-l-rhamnopyranosyl-(1 → 4)-*α*-l-rhamnopyranosyl(1 → 4)-[*α*-l-rhamnopyranosyl(1 → 2)]-*β*-d-glucopyranoside	*S. krausiana*	Rhizomes	[50]
53	26-*O*-*β*-d-Glucopyranosyl-22*α*-hydroxy-(25*R*)-furost-5-en-3*β*,26-diol-3-*O*-[4-*O*-acetyl-*α*-l-rhamnopyranosyl]-(l → 2)-[*α*-l-rhamnopyranosyl-(l → 4)]-*β*-d-glucopyranoside	*S. excelsa*	Rhizomes	[17]
54	26-*O*-*β*-d-Glucopyranosyl-22*α*-hydroxy-(25*R*)-furost-5-en-3*β*,26-diol-3-*O*-[2-*O*-acetyl-*α*-l-rhamnopyranosyl]-(l → 2)-[*α*-l-rhamnopyranosyl-(l → 4)]-*β*-d-glucopyranoside	*S. excelsa*	Rhizomes	[17]
55	26-*O*-*β*-d-Glucopyranosyl-22*α*-hydroxy-(25*R*)-furost-5-en-3*β*,26-diol-3-*O*-[3-*O*-acetyl-*α*-l-rhamnopyranosyl]-(l → 2)-[*α*-l-rhamnopyranosyl-(l → 4)]-*β*-d-glucopyranoside	*S. excelsa*	Rhizomes	[17]
56	26-*O*-*β*-d-Glucopyranosyl-(25*R*)-furostan-5-en-3*β,*17*α*-diol-3-*O*-*α*-l-rhamnopyranosyl-(l → 2)-*α*-l-rhamnopyranoside	*S. scobiniculis*	Rhizomes	[51]
57	Protogracillin	*S. riparia*	Rhizomes and roots	[20]
58	Parisaponin I	*S. riparia*	Rhizomes and roots	[20]
59	Parisyunnanoside A	*S. riparia*	Rhizomes and roots	[20]
60	Pseudoprotodioscin	*S. china* *S. trinervula* *S. menispermoides* *S. stans* *S. excelsa* *S. china* *S. nigrescens*	Roots Rhizomes and roots Rhizomes Roots Rhizomes Tubers Roots	[8] [32] [13] [16] [17] [30] [49]
61	26-*O*-*β*-d-Glucopyranosyl-(25*R*)-furostan-5,20(22)-dien-3*β*,26-diol-3-*O*-*α*-l-rhamnopyranosyl-(l → 2)-*β*-d-glucopyranoside	*S. nigrescens*	Roots	[49]
62	15-Hydroxypseudoprotodioscin	*S. china*	Tubers	[27]
63	15-Methoxypseudoprotodioscin	*S. china*	Tubers	[27]
64	23-Oxopseudoprotodioscin	*S. microphylla*	Tubers	[18]
65	26-*O*-*β*-d-Glucopyranosyl-(25*S*)-5-furosa-20(22)-en-3*β*,26-diol-3-*O*-*α*-l-rhamnopyranosyl-(l → 2)-*O*-[*α*-l-rhamnopyranosyl-(l → 6)]-*β*-d-glucopyranoside	*S. riparia*	Roots	[52]
66	Pseudoproto-pb	*S. riparia*	Rhizomes and roots	[20]
67	26-*O*-*β*-d-Glucopyranosyl-(25*R*)-5*α*-furostan-3β,22,26-triol-6-one-3-*O*-*α*-l-arabinopyranosyl-(1 → 6)-*β*-d-glucopyranoside	*S. sieboldii* *S. scobinicaulis*	Rhizomes Rhizomes and roots	[23] [28]
68	26-*O*-*β*-d-Glucopyranosyl-(25*R*)-5*α*-furostan-3*β*,22,26-triol-6-one-3-*O*-*β*-d-glucopyranosyl-(1 → 4)-[*α*-l-arabinopyranosyl-(1 → 6)]-*β*-d-glucopyranoside	*S. sieboldii*	Rhizomes	[23]
69	26-*O*-*β*-d-Glucopyranosyl-(25*R*)-5*α*-furostan-3*β*,26-diol-22-methoxyl-6-one-3-*O*-*α*-l-arabinopyranosyl-(1 → 6)-*β*-d-glucopyranoside	*S. scobinicaulis*	Rhizomes and roots	[28]
70	26-*O*-*β*-d-Glucopyranosyl-(25*R*)-5*α*-furostan-3*β*,26-diol-22-methoxyl-6-one	*S. scobinicaulis*	Rhizomes and roots	[28]
71	26-*O*-*β*-d-Glucopyranosyl-(25*S*)-5*β*-furostan-3*β*,26-diol-22*α*-methoxy-3-*O*-*β*-d-glucopyranosyl-(1 → 6)-[*β*-d-glucopyranosyl-(1 → 2)]-[*β*-d-glucopyranosyl-(1 → 4)]-*β*-d-glucopyranoside	*S. medica*	Rhizomes	[25]
72	(25*S*)-26-*O*-*β*-d-glucopyranosyl-3*β,*5*β*,22*α*-furostan-3,22,26-triol-3-*O*-*α*-l-rhamnopyranosyl-(l → 2)-*O*-*β*-d-glucopyranosyl-(l → 2)-*O*-*β*-d-glucopyranoside	*S. aspera* subsp. *mauritanica*	Roots	[10]
73	Asparagoside E	*S. aspera* subsp. *mauritanica*	Roots	[10]
74	Asparoside A	*S. aspera* subsp. *mauritanica*	Roots	[10]
75	Asparoside B	*S. aspera* subsp. *mauritanica*	Roots	[10]
76	26-*O*-*β*-d-Glucopyranosyl-(25*S*)-5*β*-furostan-3*β*,22*α*-diol-3-*O*-*α*-l-arabinopyranosyl-(l → 6)-[*β*-d-glucopyranosyl-(1 → 4)]-*β*-d-glucopyranoside	*S. officinalis*	Roots	[29]
77	26-*O*-*β*-d-Glucopyranosyl-(25*S*)-5*α*-furostan-3*β*,22*α*-diol-3-*O*-*α*-l-arabinopyranosyl-(l → 6)-[*β*-d-glucopyranosyl-(1 → 4)]-*β*-d-glucopyranoside	*S. officinalis*	Roots	[29]
78	26-*O*-*β*-d-Glucopyranosyl-(25*S*)-5*α*-furostan-3*β*,6*β,*22*α*-tetraol-3-*O*-*α*-l-arabinopyranosyl-(l → 6)-[*β*-d-glucopyranosyl-(1 → 4)]-*β*-d-glucopyrano side	*S. officinalis*	Roots	[29]
79	Sarsaparilloside B	*S. ornate*	Rhizomes and roots	[11]
80	Sarsaparilloside C	*S. ornate*	Rhizomes and roots	[11]
81	Sarsaparilloside	*S. ornate*	Rhizomes and roots	[11]
82	Δ^20(22)^-Sarsaparilloside	*S. ornate*	Rhizomes and roots	[11]
83	Riparoside A	*S. riparia*	Rhizomes and roots	[53]
84	Smilaxchinoside A	*S. china*	Tubers	[30]
85	(25*R*)-Smilaxchinoside A	*S. china* *S. riparia* *S. riparia* *S. trinervula*	Tubers Rhizomes and roots Roots Rhizomes and roots	[30] [20] [54] [32]
86	Smilaxchinoside B	*S. china*	Tubers	[30]
87	Smilaxchinoside C	*S. china* *S. riparia*	Tubers Rhizomes and roots	[30] [20]
88	Dioscoreside E	*S. trinervula*	Rhizomes and roots	[32]
89	(25*R*)-5*α*-Furostan-3*β*,26-diol-20(22)-en-6-one-26-*O*-*β*-d-glucopyranoside	*S. scobinicaulis*	Rhizomes and roots	[28]
90	(23*R*,25*R*)-5*α*-Furostan-3*β*,23,26-triol-20(22)-en-6-one-26-*O*-*β*-d-glucopyranoside	*S. scobinicaulis*	Rhizomes and roots	[28]
91	26-*O*-*β*-d-Glucopyranosyl-(25*R*)-5*α*-furostan-3*β*,26-diol-20(22)-en-6-one-3-*O*-*α*-l-arabinopyranosyl-(1 → 6)-*β*-d-glucopyranoside	*S. scobinicaulis*	Rhizomes and roots	[28]
92	(25*S*)-5*β*-Furostan-1*β*,2*β*,3*β*,5*β*,22*α*,26-hexaol-26-*O*-*β*-d-glucopyrano side	*S. aspera*	Rhizomes	[31]
93	26-*O*-*β*-d-Glucopyranosyl-(25*S*)-5*β*-furostan-1*β*,3*β*,22*α*,26-tetraol-1-*O*-*β*-d-glucopyranoside	*S. aspera*	Rhizomes	[31]
*Pregane-type saponin*				
94	Trinervuloside A	*S. trinervula*	Rhizomes and roots	[32]
95	Riparoside B	*S. riparia* *S. riparia* *S. riparia*	Rhizomes and roots Rhizomes and roots Rhizomes and roots	[20] [55] [53]
96	Timosaponin J	*S.riparia* *S. riparia*	Rhizomes and roots Rhizomes and roots	[20] [55]
97	Timosaponin K	*S. riparia*	Rhizomes and roots	[20]
98	Trinervuloside B	*S. trinervula*	Rhizomes and roots	[32]
99	Pregna-5,16-diene-3*β*-ol-20-one-3-*O*-*α*-l-rhamnopyranosyl-(l → 2)-*O*-[*α*-l-rhamnopyranosyl-(l → 4)]-*β*-d-glucopyranoside	*S. nigrescens* *S. bockii* *S. menispermoides*	Roots Roots Rhizomes	[15] [19] [33]
100	Pregna-5,16-diene-3*β*-ol-20-one-3-*O*-*α*-l-rhamnopyranosyl-(l → 4)-*α*-l-rhamnopyranosyl-(l → 4)-[*α*-l-rhamnopyranosyl-(l → 2)]-*β*-d-glucopyranoside	*S. bockii*	Roots	[19]
101	Pregna-5,16-diene-3*β*-ol-20-one-3-*O*-*α*-l-rhamnopyranosyl-(l → 2)-[*α*-l-rhamnopyranosyl-(l → 4)]-*α*-l-rhamnopyranosyl-(l → 2)-*β*-d-glucopyranoside	*S. microphylla*	Tubers	[18]
102	Pallidfloside D	*S. riparia*	Rhizomes and roots	[20]
*Cholestane-type saponin*				
103	Anguivioside XV	*S. trinervula*	Rhizomes and roots	[32]
104	Smilaxchinoside D	*S. china*	Tubers	[30]

### Pregnane-Type Saponins **94**–**102** and Others **103**–**104**

**Fig. 5:**
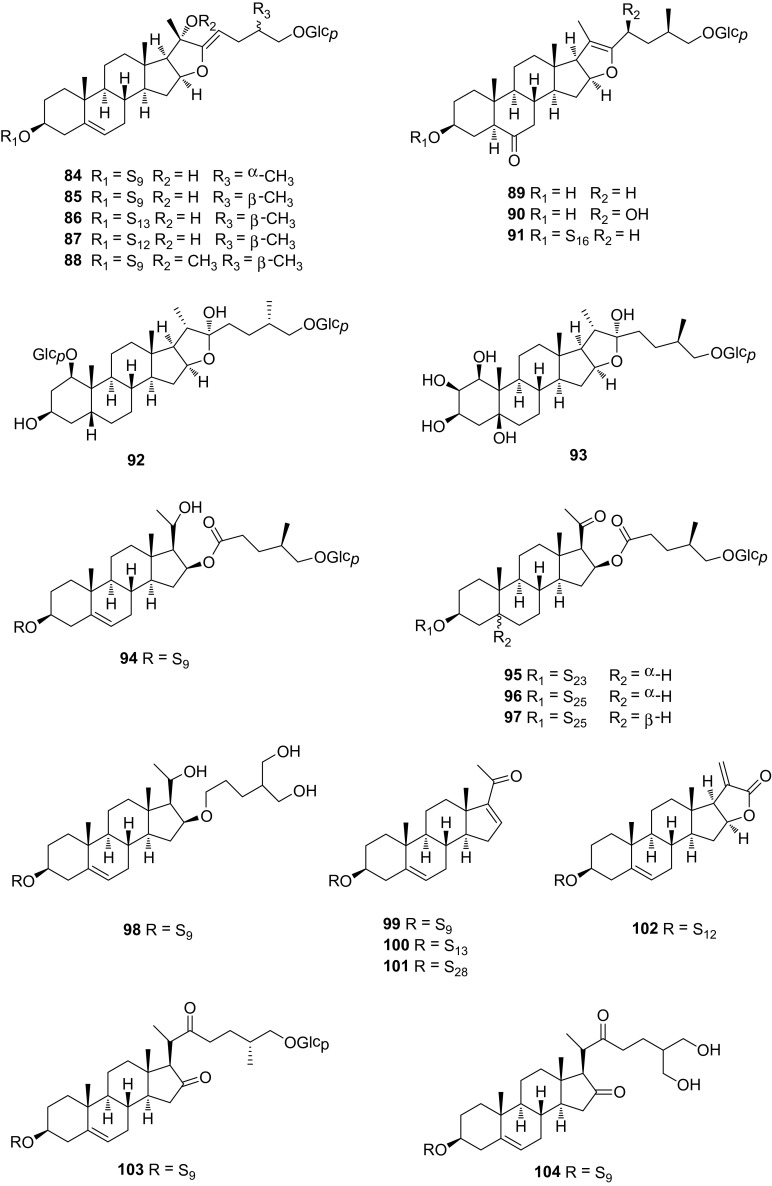
Structures of compounds **84**–**104**

Pregane-type saponins are C_21_ steroidal saponins with a sugar moiety linked to the alcoholic hydroxyl group of the sapogenin, most frequently at C-3. Compounds **94**–**98** are not real pregnane-type saponins from the perspective of biosynthetic pathway. Possibly, they are biosynthetically formed through oxidative cleavage of the double bond between C-20 and C-22 in furostane structures. Compounds **94** and **98** were isolated from the rhizomes and roots of *S. trinervula*, together with compounds **11**, **60**, **85**, **88**, and **103** [32]. Pregnane glycosides **99**–**102** were found in *S. nigrescens* [15], *S. menispermoidea* [33], *S. bockii* [19], *S. microphylla* [18], and *S. riparia* [20]. Compounds **103** and **104**, isolated from *S. trinervula* and *S. china* respectively, are belonged to cholestane-type saponins, or open chain saponins in another way of saying [34]. *S. riparia* saponins, from which compounds **18**–**22**, **57**–**59**, **66**, **85**, **87**, **95**–**97**, and **102** were identified, exhibited the synergistic effects with allopurinolin in reducing serum uric acid levels and increasing the urine uric acid level in a hyperuricemic mouse model [20]. The attenuation of hyperuricemia-induced renal dysfunction was linked to the inhibition of serum and hepatic xanthine oxidase, the down-regulation of renal mURAT1 and GLUT9, and the up-regulation of mOAT1. Structures of steroidal saponins (**94**–**104**) are shown in Fig. 5.

## Biological Activities of Steroidal Saponins

Steroidal saponins are considered to be responsible for pharmacological properties of *Smilax* species. Many pharmacological in vitro and in vivo studies revealed significant biological activities, including cAMP phosphodiesterase inhibitory, anti-fungal, cytotoxic, and anti-inflammatory activities.

**Fig. 6:**
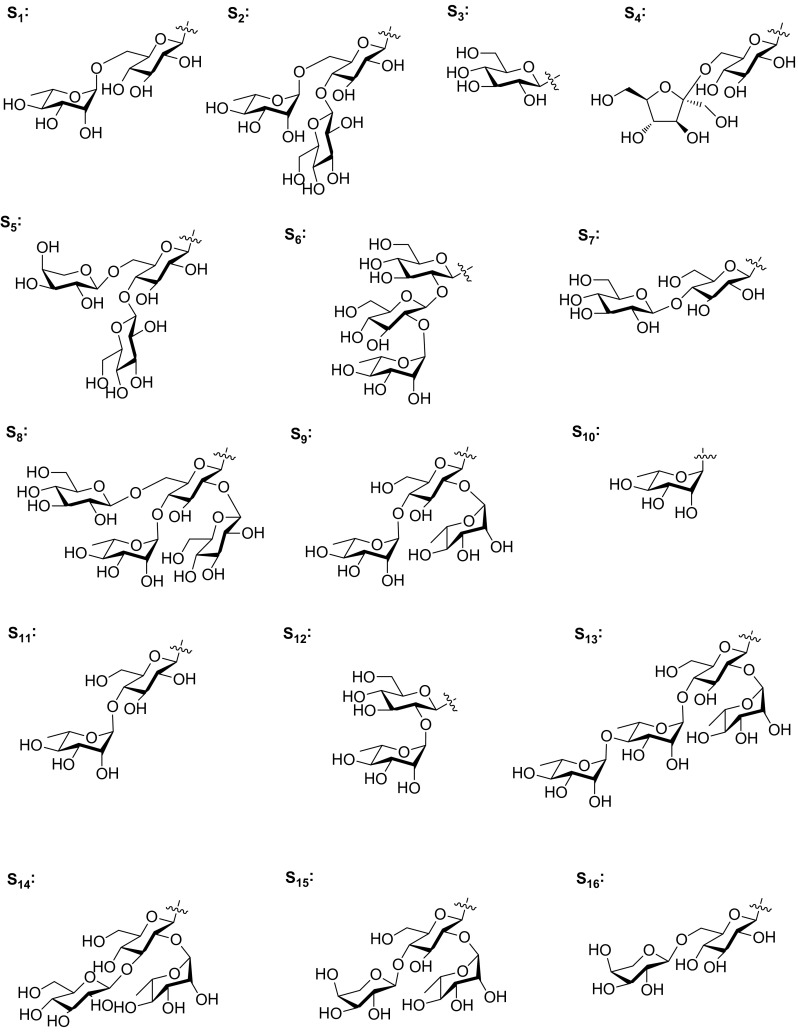
Sugar residues of S_1_–S_16_

**Fig. 7:**
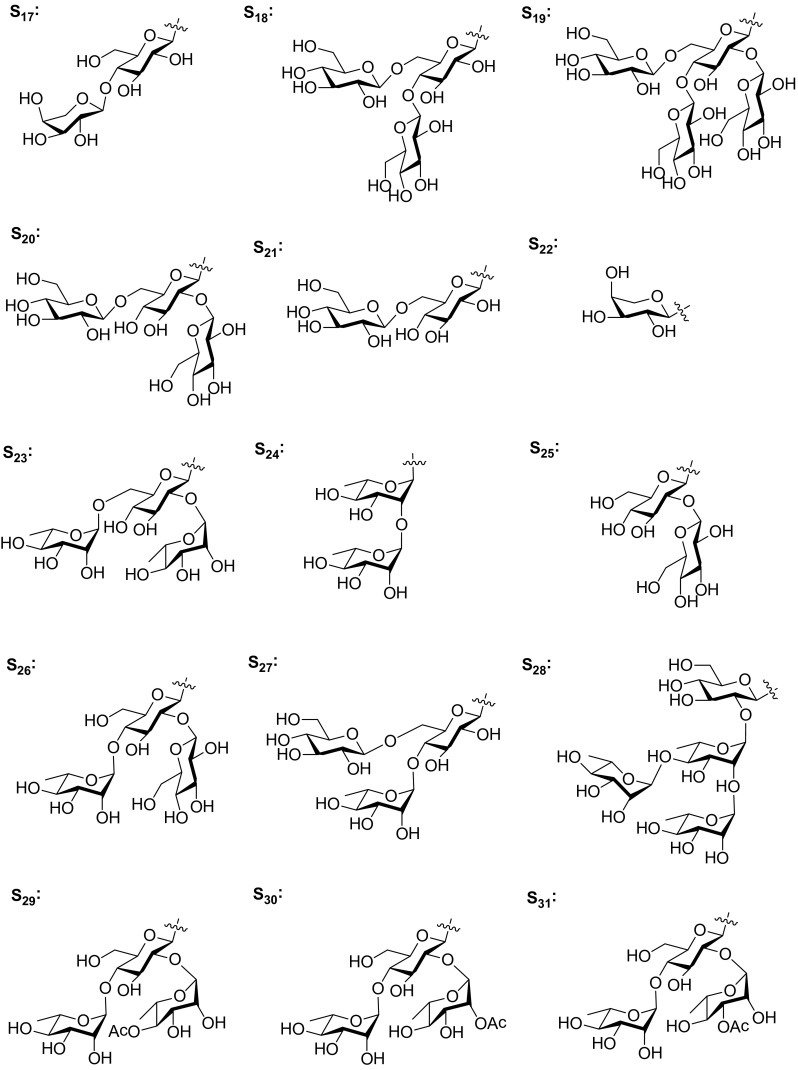
Sugar residues of S_17_–S_31_

### cAMP Phosphodiesterase Inhibitory Activity

The cAMP phosphodiesterase is an enzyme that degrades the phosphodiester bond in the second messenger molecule cAMP. It regulates the localization, duration, and amplitude of cyclic nucleotide signaling within subcellular domains. Compounds **1**, **2**, **29** and **60**, showed cAMP phosphodiesterase inhibitory activities with IC_50_ values of 102, 55, 93, and 47 μM, respectively, which were almost equal to that of positive control papaverine (IC_50_ = 30 μM) [8]. Laxogenin glycosides **34**, **35**, and isospirostanol glycoside **38** displayed cAMP phosphodiesterase inhibitory activities with IC_50_ values of 83, 34, and 32 μM, respectively. While compound **36**, with an additional hydroxyl substitution on C-27 in comparison with compound **34**, showed no obvious inhibitory activity. Furostane glycosides **67**–**68** were inactive [23].

### Antifungal Activity

C_27_ steroidal glycosides are well known for their antifungal activities [35]. Sarsasapogenin glycosides **7**, **8**, and four furostane glycosides **72**–**75**, were tested for their antifungal activity. Compound **8** showed antifungal activity against three human pathogenic species, *Candida albicans*, *C. glabrata*, and *C. tropicalis*, with minimal inhibitory concentration (MIC) values of 25, 25 and 50 μg/mL, respectively. While compounds **7** and **72**–**75** showed no obvious antifungal activity at concentration of 200 μg/mL [10]. Six smilagenin glycosides **42**–**47** and a furostane glycoside **71** were also evaluated for their antifungal activities against these three pathogenic species. Compounds **42**–**46** demonstrated moderate antifungal activity with MIC values between 12.5 and 50 μg/mL [25, 26]. With regard to structure–activity relationships between the saponin structures and antifungal activities, the following points were suggested: (1) the close F ring is essential for the anti-fungal activities. (2) The *cis*/*trans* fusion between rings A and B has no significant difference in terms of antifungal activities. (3) Steroidal saponins bearing a saccharidic chain with more than one sugar were better antifungal agents (Figs. 6, 7).

### Cytotoxicity

Spirostane glycoside **9** and four furostane glycosides **79**–**82** were evaluated for their cytotoxicities against six human cancer cells (NFF, Hela, HT29, MCF7, MM96L, and K562). Compounds **79** and **80** selectively inhibited the proliferation of the HT29 colon cancer cell lines with IC_50_ values of 4.8 and 5.0 μg/mL, respectively; while compounds **80** and **81** showed significant cytotoxicities aganist MCF7 cell lines with IC_50_ values of 9.5 and 3.4 μg/mL, respectively [11]. Compounds **24**, **25**, **28**, **38**, and **39**, were evaluated for the cytotoxicities against three human cancer cell lines (A549, LAC and Hela). Only compound **38** possessed significant cytotoxicities with IC_50_ values of 3.70, 5.70 and 3.64 μM, respectively [21]. Another cytotoxic compound is isospirostane glycoside **32**, which displayed potent cytotoxicities against the Hela and SMMC-7221 cancer cell lines with IC_50_ values of 9.73 ± 1.64 and 21.54 ± 1.64 μM, respectively [28]. The above results indicated that the hydroxyl substitutions on C-6 or C-17 of isospirostane glycosides decrease the cytotoxicities. Furostane glycoside **69** showed cytotoxicities against the Hela and SMMC-7221 cancer cell lines with IC_50_ values of 18.79 ± 1.12 and 28.57 ± 1.57 μM, respectively; while the demethylated analogue **67** and the dehydrated analogues **89**–**91** showed no obvious cytotoxicities. Additionally, the sapogenin **70** was less cytotoxicities than that of corresponding glycoside **69** [28]. Compounds **11**, **60**, **85**, **88**, **94**, **98** and **103**, were tested for their cytotoxicities against SHSY5Y, SGC-7901, HCT-116 and Lovo cell lines. Only compound **98** showed significant cytotoxicities against SGC-7901 and HCT-116 cell lines with IC_50_ values of 8.1 and 5.5 μM, respectively [32].

### Anti-inflammatory Activity

The aqueous extracts of the tubers of *S. china* showed the similar anti-inflammatory effects in vivo to that of acetylsalicylic acid (200 mg/kg, i.g.) [36]. Sieboldogenin (**33**) showed significant lipoxygenase inhibition activity with IC_50_ value of 38 μM. It also exhibited significant inhibition on carrageenan-induced hind paw oedema at the doses of 10 and 50 mg/kg [22]. Compounds **13**, **14**, **16**, **48**, **84**–**87**, and **104** inhibited the lipoposaccharide (LPS) induced prostaglandin E_2_ (PGE_2_) production in murine peritoneal macrophages by 81.5, 81.7, 76.5, 82.5, 76.1, 59.1, 78.5, 75.9, and 82.0%, respectively, at the concentration of 10 μM. These nine compounds also moderately inhibited the tumor necrosis factor α (TNFα) production on LPS stimulated murine peritoneal macrophages [30].

## Prospects

The plants of the genus *Smilax* are widely spread in China. Their medical use for the treatment of inflammation and rheumatism has a long history in folk China. Previous studies on chemical constituents of *Smilax* sp. yielded diversified steroidal saponin. However, the biological activities studies of these isolated steroidal saponins lag behind, especially in anti-inflammatory related activities.
